# Serum vitamin D level is inversely associated with liver fibrosis in post Kasai’s portoenterostomy biliary atresia patients living with native liver

**DOI:** 10.1371/journal.pone.0218896

**Published:** 2019-06-26

**Authors:** Chia-Huei Peng, Hung-Chang Lee, Chuen-Bin Jiang, Cheng-Kai Hsu, Chun-Yan Yeung, Wai-Tao Chan, Szu-Wen Chang, Shu-Chao Weng

**Affiliations:** 1 Department of Pediatric Gastroenterology, Hepatology and Nutrition, MacKay Children’s Hospital, Taipei, Taiwan; 2 Department of Nephrology, Chang Gung Memorial Hospital, Keelung, Taiwan; Texas A&M University, UNITED STATES

## Abstract

**Objective:**

This study aims to investigate the association of serum vitamin D (VD) levels with the severity of liver fibrosis (LF) in post Kasai’s portoenterostomy biliary atresia (PKBA) patients living with their native liver.

**Methods:**

In this cross-sectional study, carried out in a tertiary Children’s Hospital in Taipei, Taiwan, PKBA patients living with their native liver were enrolled. Liver biochemistry data, serum 25-hydroxyvitamin D (25-OHVD), acoustic radiation force impulse (ARFI), and scores of Pediatric Quality of Life questionnaire (PedsQL) were collected.

**Results:**

All the enrolled 33 PKBA patients (36.4% males), aged 1–23 years, possessed 25-OHVD less than 30ng/ml. An inverse correlation was detected between serum 25-OHVD and ARFI (r^2^ = 0.175; *p* = 0.024). We selected a cutoff value of 23ng/mL to divide PKBA patients into two groups, as the *p*-value was the most significant at this point when comparing the median ARFI of two groups (*p* = 0.003). Ten (30.3%) had 25-OHVD≥23ng/ml (HVD group), whereas 23(69.7%) had 25-OHVD<23ng/ml (LVD group). HVD group had lower ARFI (1.13m/s vs. 1.52m/s, *p* = 0.003), lower aspartate transaminase (AST) (29U/L vs. 64U/L, *p* = 0.033), and higher scores of self-reported health-related quality of life in psychosocial functioning (86.7 vs. 77.1, *p* = 0.047) than LVD group.

**Conclusion:**

VD levels are inversely associated with severity of LF in PKBA patients with native liver.

## Introduction

Biliary atresia (BA) is the most common cause of liver cirrhosis in children. BA occurs in approximately 1.23 of 10,000 live births in Taiwan and 0.56 of 10,000 live births in European countries [1, 2]. Kasai’s portoenterostomy (KPE) is the first line therapy to reconstruct the obliterated bile ducts and restore bile flow. The introduction of KPE made long-term survival possible. In Japan, the 10-year native liver survival rate is 52.8%, while in Taiwan, the infant stool card screening program for BA started in 2002 allowed earlier KPE and yielded improvement of 5-year-survival rate with native liver (NL) from 56% to 89%[3, 4]. However, more than 60% of patients experienced complications such as ascending cholangitis (36%), portal hypertension (40%), gastrointestinal bleedings (25%) and hepatocellular carcinoma [5, 6]. Only 30% of the patients with BA living with NL had no clinical complications of chronic liver disease (CLD), and only 2–11% had absence of surgical complications and normal laboratory indices [6, 7]. Liver transplantation (LT) is considered in those who fail to clear jaundice by KPE or with progressive liver cirrhosis. With growing experience and advanced techniques of pediatric LT, the 5-year recipient survival rate is 82–98%[3].

Previously, it was believed that vitamin D (VD) deficiency is mainly caused by bile acid-related lipid malabsorption in cholestatic liver diseases, however, recent studies showed that both cholestatic and non-cholestatic liver diseases share mutual characteristic of VD deficiency [8]. In addition, VD deficiency has been linked to various adverse outcomes of chronic liver diseases in adults. In pediatric studies, serum VD levels are inversely associated with non-alcoholic steatohepatitis (NASH) and fibrosis in children with non-alcoholic fatty liver disease (NAFLD)[9]. Nevertheless, little research has been done to explore the relationship between VD status and the severity of liver fibrosis (LF) in post Kasai’s portoenterostomy BA (PKBA) patients. The aim of the present study was to investigate the association of serum VD levels with the severity of LF in PKBA patients living with their NL.

## Materials and methods

### Subjects

PKBA patients were consecutively recruited in this cross-sectional study between October, 2016 to March, 2018. The setting was a tertiary children’s hospital in Taipei, Taiwan. Grown-up patients who maintained regular hospital visits in MacKay Children’s Hospital were also enrolled in the study. As pure VD oral supplements were not introduced into Taiwan until 2014, the PKBA patients in our institute were encouraged to have more sun exposure, but most of them did not receive routine VD supplements at the time of study enrollment. Two patients taking vitamin and/or mineral supplements and/or medications (polyvitamin, calcitriol, calcium carbonate) known to influence serum 25-hydroxyvitamin D (25-OHVD) status were excluded from the study. Patients who had received LT were also excluded from the study. None of the enrolled patients used VD supplements upon recruitment.

The study was approved by the Institutional Review Board of the MacKay Memorial Hospital and the approved number was 17MMHIS051; the informed consents were obtained from the parents or legal guardians of the patients or the patients themselves who aged over 18 years.

### Demographics and laboratory assessment

Age, gender, month-old when patients received KPE (before or after 2-month-old), and hospitalization frequency due to acute cholangitis were recorded. Biochemical parameters including aspartate transaminase (AST), alanine transaminase (ALT), gamma glutamyl transferase (rGT), direct bilirubin, total bilirubin, and 25-OHVD were obtained. The levels of 25-OHVD were measured with chemiluminescent method by Diasorin. Currently, the normal values of serum 25-OHVD are still in debate, while generally the suggested values range from 10 to 40ng/mL [10–12]. In this study, VD deficiency is defined as serum 25-OHVD levels lower than 20ng/mL and VD insufficiency is defined as serum levels between 20 and 30ng/mL.

### Acoustic radiation force impulse (ARFI)

All the participants were studied with a 6-MHz probe on an Acuson S3000 US machine with a Virtual Touch Tissue Quantification (VTQ) mode for ARFI elastography (Siemens Healthcare, Erlangen, Germany). ARFI measures the shear wave speed and was regarded as the marker of elasticity in the examined tissue (expressed in meter/second). ARFI values and blood tests were obtained no more than three months apart. The ARFI values were measured independently by four pediatric gastroenterologists with over 15 years of experience doing abdominal ultrasounds and at least 1 year of experience using the ARFI modality. The operator selected a rectangular region of the right liver through the intercostal space, avoiding bile ducts and blood vessels, with the size of 1cm x 0.6cm, 4 cm to 6cm from the cutaneous surface. Patients were examined in the supine position, with their right arm in maximum abduction. Patients were asked to breathe smoothly or slightly holding their breath during the measurements of ARFI. Three measurements were made and the mean value was calculated.

### Pediatric quality of life questionnaire (PedsQL)

The PedsQL Measurement Model is a reliable and valid modular approach to measuring health-related quality of life (HRQoL) in children and adolescents with acute and chronic health conditions. It is brief (contains 23 items), practical (takes less than 4 minutes to complete), developmentally appropriate (tailored for different age groups), and is translated into multiple languages. The 23 items are categorized into 4 multidimensional scales, as physical functioning (8 items), emotional functioning (5 items), social functioning (5 items), and school functioning (5 items). The 3 summary scores are total scale score (23 items), physical health summary score (8 items), and psychosocial health summary score (15 items). The interpretation of the questionnaire was made after transformation and calculation according to the PedsQL scoring procedure. The higher the score, indicates better HRQoL. Our study team was authorized to use PedsQL questionnaire to assess the quality of life of the participants. The questionnaire was distributed to the participants and their parents either by postal mails or in person during their hospital visits.

### Statistical analysis

Descriptive statistics were expressed as median (interquartile range, IQR). Discrete variables were presented as frequency and percentage. Differences in continuous variables between the 2 groups were analyzed by Mann Whitney U test. Differences in categorical variables between the 2 groups were compared by Fisher’s exact test. The association of LF with serum 25-OHVD was detected using a linear regression model.

Statistical analysis was performed with the Statistical Package for the Social Sciences (SPSS) version 21.0 (SPSS, Inc., USA). All statistical tests were two-tailed, and a *p*-value < 0.05 was considered statistically significant.

## Results

### Grouping

**Fig 1:**
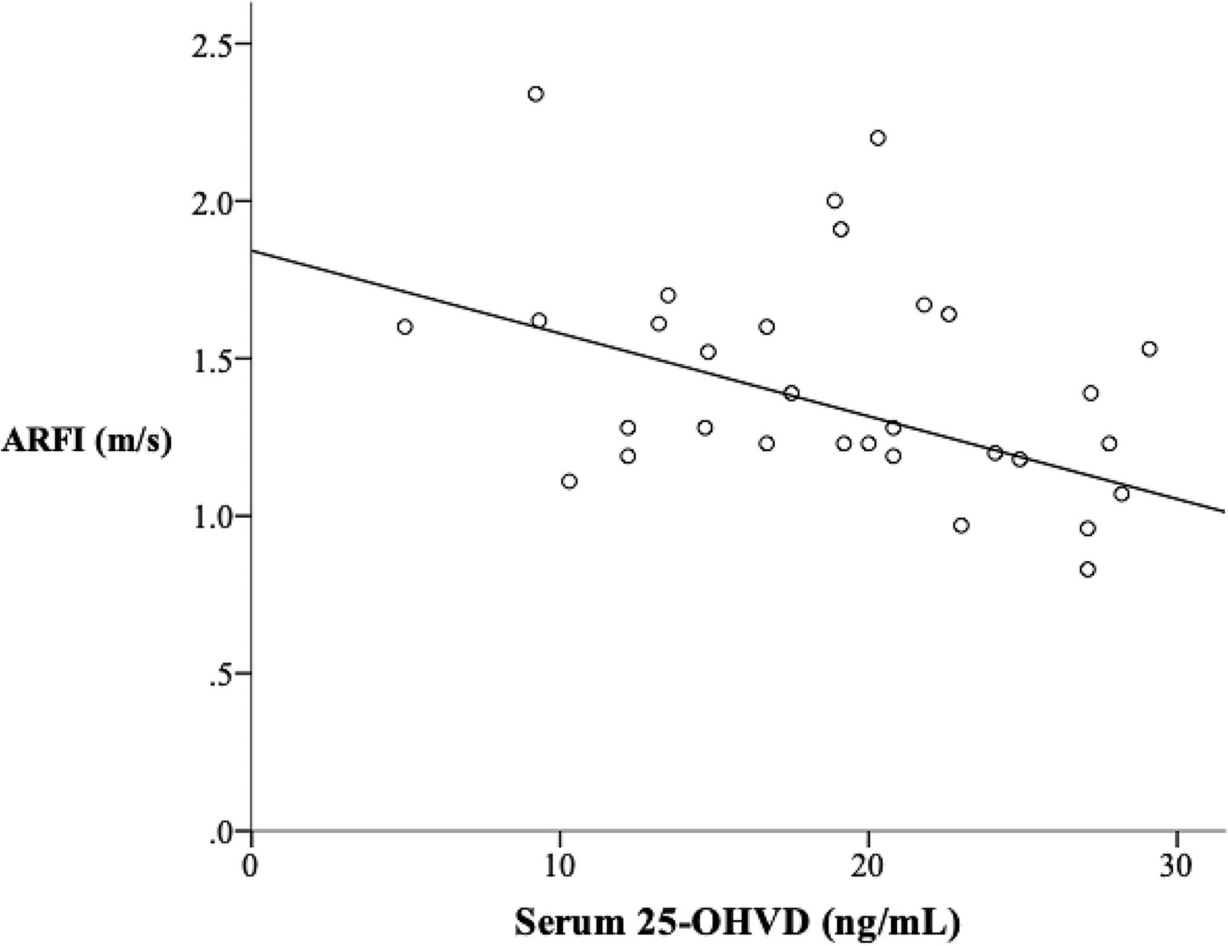
Serum 25-OHVD had a negative correlation with ARFI (r^2^ = 0.175; *p* = 0.024). Abbreviations: 25-OHVD: 25-hydroxyvitamin D; ARFI: Acoustic radiation force impulse.

A total of 33 patients (36.4% males) aged 1–23 years were enrolled into our study. All the patients’ 25-OHVD were less than 30ng/ml. According to the scatter diagram, there was an inverse association between serum 25-OHVD and ARFI values (r^2^ = 0.175; *p* = 0.024)(Fig 1). We set multiple cutoff values of 25-OHVD dividing the patients into two groups (higher 25-OHVD and lower 25-OHVD groups), compared the medians of ARFI of the two groups with Mann-Whitney U test and calculated the *p*-values (Table 1). The *p*-value was the most significant when the cutoff value of 25-OHVD was 23ng/mL (*p* = 0.003). Of the 33 patients, 10 (30.3%) had 25-OHVD≥ 23ng/ml, whereas 23(69.7%) had 25-OHVD<23ng/ml. We name the group with 25-OHVD≥ 23ng/ml as higher VD level group (HVD group), and the group with 25-OHVD<23ng/ml as lower VD group (LVD group).

**Table 1:** Different cutoff values of 25-OHVD and corresponding *p*-values when comparing the medians of ARFI of the two groups.

25-OHVD ng/mL as cutoff value	*p*-value	25-OHVD ng/mL as cutoff value	*p*-value
10	0.080	18	0.180
11	0.061	19	0.062
12	0.061	20	0.033
13	0.330	21	0.040
14	0.100	22	0.011
15	0.138	23	0.003
16	0.140	24	0.009
17	0.180	25	0.078

Abbreviations: 25-OHVD: 25-hydroxyvitamin D; ARFI: Acoustic radiation force impulse

### Age and gender

**Fig 2:**
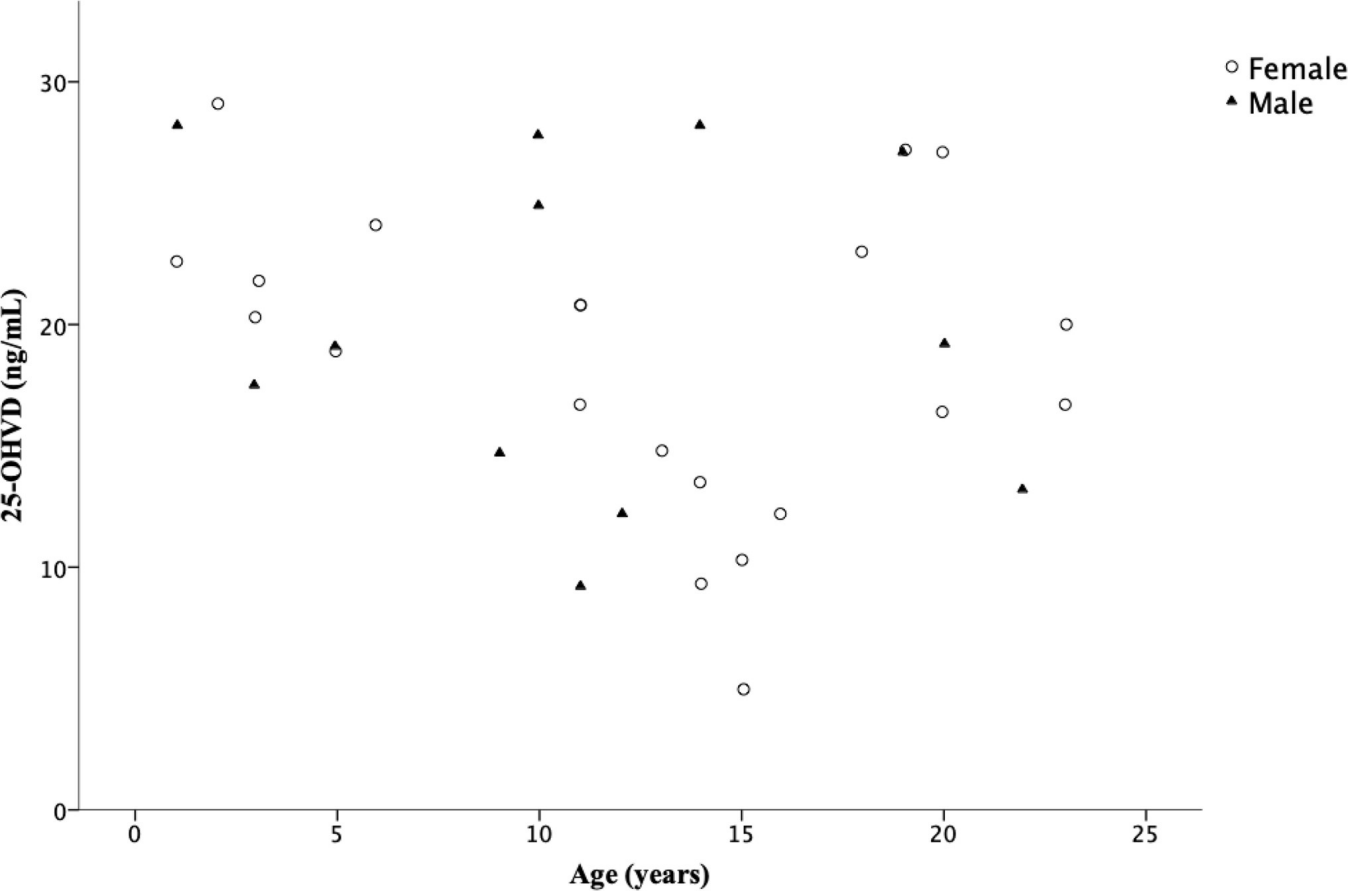
Relation between age in years and serum 25-OHVD (ng/mL) concentrations stratified by gender. Abbreviations: 25-OHVD: 25-hydroxyvitamin D.

There were 12 males and 21 females among the 33 PKBA patients. LVD group consisted of 7 males and 16 females, and HVD group consisted of 5 males and 5 females. There was no age difference between LVD and HVD groups (LVD: 12.0 (5.0–16.0), HVD: 12.0 (5.0–19.0), *p* = 0.814). There was no correlation between age and 25-OHVD in our patients (r^2^ = 0.06, *p* = 0.17); moreover, neither of the gender showed decline of 25-OHVD with increase of age (female: r^2^ = 0.093, *p* = 0.167; male: r^2^ = 0.014, *p* = 0.725). (Fig 2).

### Serum biochemical parameters

The median AST value is higher in LVD group than HVD group (64 vs. 29, *p* = 0.033). The medians of other measurements including ALT, total bilirubin, direct bilirubin and rGT were also higher in LVD group comparing to those in HVD group, although there was no statistical significances (Table 2).

**Table 2:** Demographic and liver function tests of PKBA patients.

Parameter	All patients (n = 33)	25-OHVD < 23 (n = 23)	25-OHVD ≥ 23 (n = 10)	*p*-value
Age (years)	12.0 (5.5–18.5)	12.0 (5.0–16.0)	12.0 (5.0–19.0)	0.814
Male, No. (%)	12 (36.4)	7 (30.4)	5 (50)	0.433
Weight for age				0.054
5–15%	2	2 (8.7%)	0 (0)	
15–50%	7	2 (8.7%)	5 (50%)	
50–85%	15	11 (47.8%)	4 (40%)	
85–97%	9	8 (34.8%)	1 (10%)	
AST (U/L)	48 (28.5–94.5)	64 (34.5–132.75)	29 (21–46)	0.033
ALT (U/L)	78 (30–123.5)	98 (39.25–202)	32 (17–92)	0.063
Total bilirubin (mg/dL)	0.7 (0–1.45)	1.4 (1.1–1.9)	0.7 (0.7–0.7)	0.259
Direct bilirubin (mg/dL)	0.3 (0.2–0.9)	0.3 (0.2–0.9)	0.1 (0.1–0.1)	0.166
r-GT (IU/L)	103 (50–207)	121.5 (71.75–235)	64 (31–124.5)	0.139

Data are expressed as median (interquartile range) or number (percentage).

Abbreviations: 25-OHVD: 25-hydroxyvitamin D; ALT: alanine transaminase; AST: aspartate transaminase; PKBA: post Kasai’s portoenterostomy biliary atresia; rGT: gamma glutamyl transferase

### ARFI values and PedsQL scores

**Fig 3:**
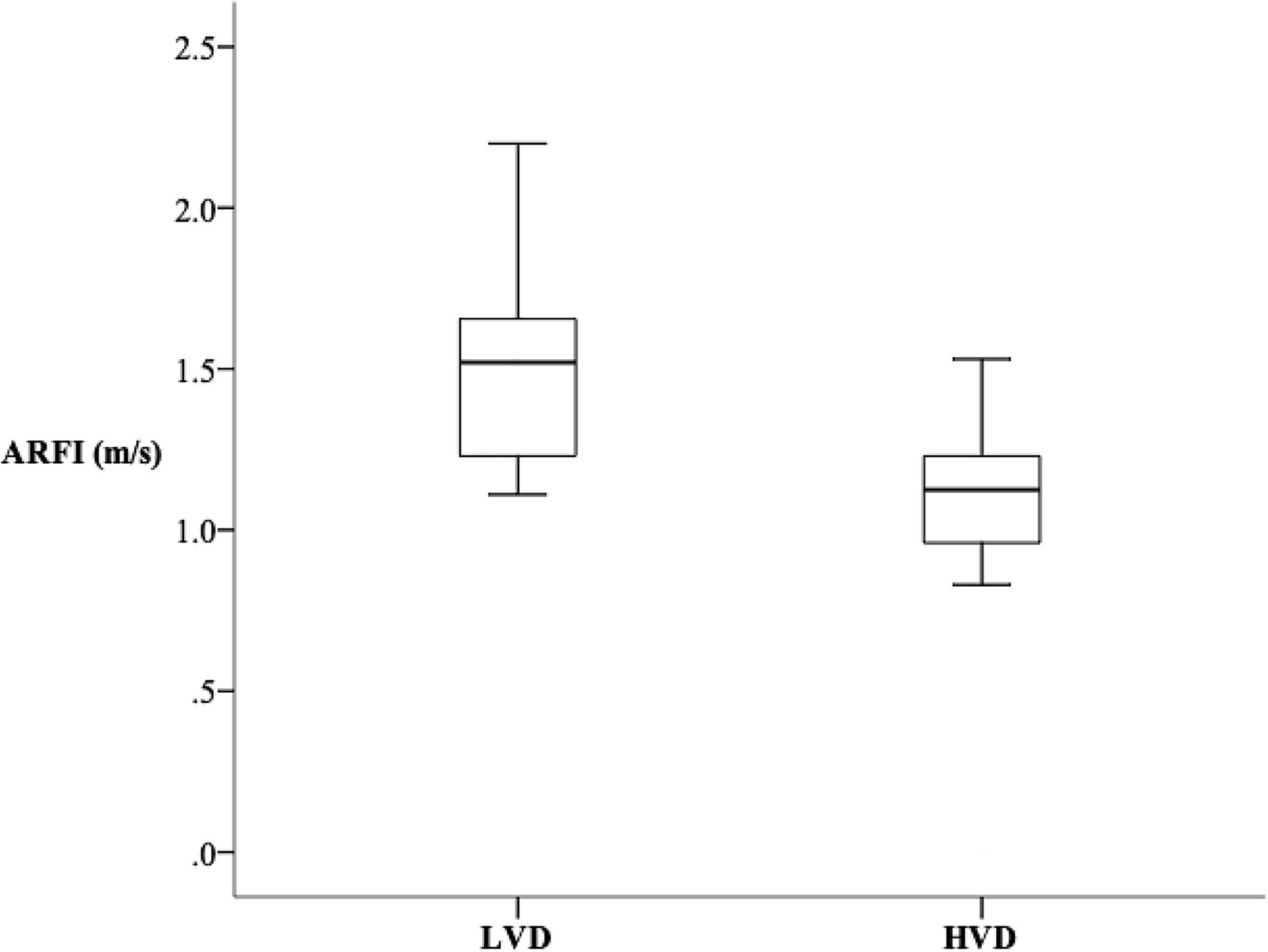
Patients in LVD group had higher ARFI values than those in HVD group (1.52m/s vs. 1.13m/s, *p* = 0.003), indicating patients with lower serum 25-OHVD levels had more severe liver fibrosis. Abbreviations: LVD: lower vitamin D level; ARFI: Acoustic radiation force impulse; HVD: higher vitamin D level; 25-OHVD: 25-hydroxyvitamin D.

25-OHVD had a negative correlation with ARFI (r^2^ = 0.175; *p* = 0.024) in Fig 1. Patients in LVD group had higher ARFI values (1.52 vs. 1.13, *p* = 0.003) than those in HVD group, indicating patients with lower 25-OHVD had more severe LF (Fig 3). The psychosocial health summary score is significantly lower in LVD group compared to HVD group (77.1 vs. 86.7, *p* = 0.047) (Table 3).

**Table 3:** Serum 25-OHVD, ARFI values, and PedsQL scores of PKBA patients.

Parameter	25-OHVD < 23 (n = 23)	25-OHVD ≥ 23 (n = 10)	*p*-value
25-OHVD (ng/mL)	16.7 (12.2–20.0)	27.2 (24.7–28.2)	<0.001
ARFI (m/s)	1.52 (1.23–1.67)	1.13 (0.93–1.27)	0.003
Physical score	92.2 (67.2–97.7)	90.6 (75.0–96.9)	0.877
Psychosocial score	77.1 (77.3–87.1)	86.7 (80.8–93.8)	0.047
Total score	80.6 (73.4–90.8)	87.5 (81–91.3)	0.207
Proxy physical score	90.6 (71.9–96.9)	85.9 (70.3–95.3)	0.739
Proxy psychosocial score	81.7 (68.3–91.7)	71.7 (62.9–81.3)	0.321
Proxy total score	76.0 (67.4–87.0)	83.7 (71.5–91.3)	0.482

Data are expressed as median (interquartile range).

Abbreviations: 25-OHVD: 25-hydroxyvitamin D; ARFI: Acoustic radiation force impulse; PedsQL: Pediatric Quality of Life questionnaire; PKBA: post Kasai’s portoenterostomy biliary atresia

## Discussion

In this single center study, we showed that low VD status is extremely common in PKBA patients with NL in different ages. All our patients had 25-OHVD below 30ng/mL, with insufficiency in 17 patients, and deficiency in 16 patients. It is not surprising to see such universe inadequacy of serum 25-OHVD in our patient category, in which most of them have CLD. Our study observed an inverse association between serum 25-OHVD and severity of LF indicated by ARFI values. After respective analyses of multiple cutoff values dividing our patients into two groups of 25-OHVD statuses, we found that the two groups exhibited the most significant difference when the cutoff value was 23ng/mL. The median ARFI value in LVD group was 1.52m/s, which corresponded to F2 (moderate fibrosis) in METAVIR scoring system; and the median ARFI value in HVD group was 1.13m/s, which corresponded to F0 (no fibrosis).

Additionally, we found that LVD group displayed a higher AST level than HVD group (64IU/L vs. 29IU/L, *p* = 0.033), which probably suggested a more severe hepatic inflammation in LVD group. Self-reported HRQoL summed a lower score of psychosocial functioning in LVD group; it could be postulated that lower VD status reflects poorer liver condition and is prone to relatively impaired psychosocial function, or that lower VD status is connected to depressive disorder to some degree. However, the other HRQoL dimensions did not show concordant results as psychosocial functioning between the two groups.

VD deficiency is a global health problem that had received increasing attention recently. Normal values of serum 25-OHVD are still in debate. According to the Institute of Medicine of the National Academies in the United States, VD concentration of 20ng/mL is largely sufficient [10]. However, the Endocrine Society recommends higher thresholds, above 30ng/mL, as sufficient. Because evidence show that VD levels above 20ng/ml prevent rickets and osteomalacia; while level above 30ng/ml maximizes 25-OHVD’s effect on calcium, bone and muscle metabolism, and may have additional benefits in reducing cancers, autoimmune diseases, type 2 diabetes, cardiovascular disease, and infectious diseases. In this study, we define VD deficiency as serum 25-OHVD levels lower than 20ng/mL and insufficiency as levels between 20 and 30ng/mL [11, 12].

According to recent survey, VD deficiency accounts for 8–12% in healthy white infants and toddlers [13, 14]. In southern China, VD deficiency is 10.8% and insufficiency is 39% among children [15]. In northern Taiwan, among healthy 3–18 year-olds, 51% have VD deficiency, and 40% have VD insufficiency [16]. Beyond regulation of calcium and bone homeostasis, VD has an emerging role in proliferation, differentiation, inflammation, and immunomodulation. VD2 is synthesized from food, whereas VD3 is mainly synthesized in the epidermis after sun exposure [17]. Bile acids are requisite for gastrointestinal absorption of dietary sources of VD. VD2 and D3 are bound to VD binding protein (VDBP) in the blood circulation, undergo hydroxylation in the liver forming 25-OHVD, then hydroxylate mainly in the kidneys into 1,25(OH)_2_D [18]. 1,25(OH)_2_D is also produced by immune cells in a paracrine fashion, which participates in immunomodulation [19]. 1,25(OH)_2_D, the biologic active form of VD, exerts its diverse functions by binding with VD receptors (VDR), which are expressed in many different tissues [14]. It has been reported that low VD status is a common feature in different types of liver diseases [14]. Previously, it was believed that VD deficiency is attributed to bile acid-related lipid malabsorption in cholestatic liver diseases; however, recent studies show that both cholestatic and non-cholestatic liver diseases share mutual characteristic of VD deficiency [8]. In adult studies, serum 25-OHVD levels correlate inversely with liver disease severity in cirrhosis [8, 14]. Possible mechanisms include malnutrition, decreased sun exposure, reduced synthesis of VDBP, and impaired bile acid excretion. Moreover, advanced studies discover a causal association between VD deficiency and LF. VDR is abundant in hepatic stellate cells (HSCs); HSCs are the major effective cells in the wound healing process when encounters liver injury, engaging in deposition of extracellular matrix proteins. In vitro study show that activation of VDR in HSCs strongly antagonize the TGFβ signaling pathway, which is the most potent pro-fibrogenic pathway in liver [20]. In addition, preclinical studies have demonstrated that systemic administration of a VDR ligand (calcitrol or calcipotriol) effectively inhibited liver fibrogenesis in standard mouse models of liver injury [21]. Therefore, maintaining sufficient VD levels is postulated to inhibit the process of LF.

Fat-soluble vitamin (FSV) deficiency is extremely common in pre-KPE BA patients. VD deficiency alone accounts for 88–98% of pre-KPE BA patients, whereas nearly half of the patients present with two or more FSV deficiency [13, 22]. Even with successful KPE and oral supplementation of FSV, the prevalence of at least one FSV insufficiency in PKBA infants is 55% throughout six months after KPE; and the prevalence of FSV insufficiency in PKBA infants with persistent cholestasis six months after KPE is 100%, 80%, 50%, and 45%, respectively, for vitamins A, D, E, and K [23]. Bile acid secretion in the liver does not reach normal levels until 6 to 12 months after KPE is probably the main reason [22]. In addition, many studies show unsatisfactory repletion of serum 25-OHVD with enteral VD supplementations in PKBA patients, likely owing to inadequate intraluminal bile acids to solubilize orally administered VD [13, 23–25]. Many experts suggest higher doses of oral VD or parenteral VD supplements to overcome this difficulty. Interestingly, the data from our study revealed that, even years after KPE, and despite clearance of jaundice, VD deficiency remains a generalized issue in PKBA patients. This supports the current understanding that multiple factors contribute to VD deficiency instead of inadequate intraluminal bile acids alone, and highlights the necessity of persistent FSV supplementation in PKBA patients. In our institute, we now treat PKBA patients with 25-OHVD<12ng/mL with oral VD3 4000IU per day, 12ng/mL≤25-OHVD<20ng/mL with 3000IU per day, 20ng/mL≤25-OHVD<30ng/mL with 2000IU per day, and 25-OHVD≥30ng/mL with 600-800IU per day. The oral VD dosage should be tailored according to sequential serum 25-OHVD levels monitored at least once a year.

Notwithstanding the considerable literature on the association of VD status and various liver diseases, this is the first study to demonstrate the association between VD status and LF in PKBA patients living with their NL. However, a series of limitations should be considered. First, this is a cross-sectional study thus cannot provide causation. Second, the sample size was small due to disease rarity, and it did contribute to reduced power of the statistics. Third, the lack of information such as the type of diet, duration of sun exposure, and the lack of serum values of VDBP does not allow us to exclude these possible confounders. Fourth, the most accurate way to evaluate LF severity is liver histology, but we substituted with ARFI, a non-invasive method instead. Fifth, the patients’ age widely ranged from 1 to 23 years old. One study (participants aging 6 to14 year-old) showed that girls had a 4% decline in serum 25-OHVD levels for each increasing year of age [26]. However, in our study, we found no correlation between age and 25-OHVD (r^2^ = 0.06, *p* = 0.17); neither of the gender showed decline of 25-OHVD with increase of age (female: r^2^ = 0.093, *p* = 0.167; male: r^2^ = 0.014, *p* = 0.725).

Hopefully in the near future, we can determine the appropriate dosage of (enteral and parenteral) VD supplementation for PKBA patients, and elucidate a causal relationship between VD status and LF in PKBA patients, by showing decrease in ARFI values following serum increment of 25-OHVD in a larger-scale, longitudinal cohort study. As the majority of PKBA patients with NL possess CLD, it is valuable to improve their quality of life and reduce complications of CLD. If we can conclude that reaching a specific serum 25-OHVD level halts the process of LF in PKBA patients, better quality of life may be achieved, and LT may be avoided or postponed. Whether simply keeping serum 25-OHVD above 23ng/mL is of clinical benefits, or higher levels are even better for these patients, is also an issue prompting further investigations.

## Conclusion

This study emphasizes the universal inadequacy of VD in PKBA patients with NL (ages range from 1 to 23 year-old and at jaundice-free phase), and the importance of monitoring their VD status. We detected a significant inverse association between 25-OHVD levels and severity of LF measured by ARFI values. Patients with serum 25-OHVD levels equal or above 23ng/mL had less severe LF than patients with serum 25-OHVD levels below 23ng/mL. Patients with higher 25-OHVD levels also had significantly lower AST levels and better self-reported HRQoL in psychosocial functioning.

## Supporting information

S1 File Raw data used for this manuscript. (XLSX)

## References

[1] Lin YC, Chang MH, Liao SF, Wu JF, Ni YH, Tiao MM, et al Decreasing rate of biliary atresia in Taiwan: a survey, 2004–2009. Pediatrics. 2011;128(3):e530–6. Epub 2011/08/30. 10.1542/peds.2011-0742 . 21873702

[2] Tiao MM, Tsai SS, Kuo HW, Chen CL, Yang CY. Epidemiological features of biliary atresia in Taiwan, a national study 1996–2003. Journal of gastroenterology and hepatology. 2008;23(1):62–6. Epub 2007/08/30. 10.1111/j.1440-1746.2007.05114.x . 17725591

[3] Kasahara M, Umeshita K, Sakamoto S, Fukuda A, Furukawa H, Uemoto S. Liver transplantation for biliary atresia: a systematic review. Pediatric surgery international. 2017;33(12):1289–95. Epub 2017/10/07. 10.1007/s00383-017-4173-5 . 28983725

[4] Lien TH, Chang MH, Wu JF, Chen HL, Lee HC, Chen AC, et al Effects of the infant stool color card screening program on 5-year outcome of biliary atresia in Taiwan. Hepatology (Baltimore, Md). 2011;53(1):202–8. Epub 2010/12/09. 10.1002/hep.24023 . 21140377

[5] Bijl EJ, Bharwani KD, Houwen RH, de Man RA. The long-term outcome of the Kasai operation in patients with biliary atresia: a systematic review. The Netherlands journal of medicine. 2013;71(4):170–3. Epub 2013/06/01. . 23723110

[6] Lee WS, Ong SY, Foo HW, Wong SY, Kong CX, Seah RB, et al Chronic liver disease is universal in children with biliary atresia living with native liver. World journal of gastroenterology. 2017;23(43):7776–84. Epub 2017/12/07. 10.3748/wjg.v23.i43.7776 29209118 PMC5703937

[7] Ng VL, Haber BH, Magee JC, Miethke A, Murray KF, Michail S, et al Medical status of 219 children with biliary atresia surviving long-term with their native livers: results from a North American multicenter consortium. The Journal of pediatrics. 2014;165(3):539–46.e2. Epub 2014/07/13. 10.1016/j.jpeds.2014.05.038 25015575 PMC4144331

[8] Malham M, Jorgensen SP, Ott P, Agnholt J, Vilstrup H, Borre M, et al Vitamin D deficiency in cirrhosis relates to liver dysfunction rather than aetiology. World journal of gastroenterology. 2011;17(7):922–5. Epub 2011/03/18. 10.3748/wjg.v17.i7.922 21412501 PMC3051142

[9] Nobili V, Giorgio V, Liccardo D, Bedogni G, Morino G, Alisi A, et al Vitamin D levels and liver histological alterations in children with nonalcoholic fatty liver disease. European journal of endocrinology. 2014;170(4):547–53. Epub 2014/01/15. 10.1530/EJE-13-0609 . 24412930

[10] Ross AC, Manson JE, Abrams SA, Aloia JF, Brannon PM, Clinton SK, et al The 2011 report on dietary reference intakes for calcium and vitamin D from the Institute of Medicine: what clinicians need to know. The Journal of clinical endocrinology and metabolism. 2011;96(1):53–8. Epub 2010/12/02. 10.1210/jc.2010-2704 21118827 PMC3046611

[11] Munns CF, Shaw N, Kiely M, Specker BL, Thacher TD, Ozono K, et al Global Consensus Recommendations on Prevention and Management of Nutritional Rickets. The Journal of clinical endocrinology and metabolism. 2016;101(2):394–415. Epub 2016/01/09. 10.1210/jc.2015-2175 26745253 PMC4880117

[12] Holick MF, Binkley NC, Bischoff-Ferrari HA, Gordon CM, Hanley DA, Heaney RP, et al Evaluation, treatment, and prevention of vitamin D deficiency: an Endocrine Society clinical practice guideline. The Journal of clinical endocrinology and metabolism. 2011;96(7):1911–30. Epub 2011/06/08. 10.1210/jc.2011-0385 . 21646368

[13] Ng J, Paul A, Wright N, Hadzic N, Davenport M. Vitamin D Levels in Infants With Biliary Atresia: Pre- and Post-Kasai Portoenterostomy. Journal of pediatric gastroenterology and nutrition. 2016;62(5):746–50. Epub 2015/12/15. 10.1097/MPG.0000000000001074 . 26655939

[14] Konstantakis C, Tselekouni P, Kalafateli M, Triantos C. Vitamin D deficiency in patients with liver cirrhosis. Annals of gastroenterology. 2016;29(3):297–306. Epub 2016/07/02. 10.20524/aog.2016.0037 27366029 PMC4923814

[15] Guo Y, Ke HJ, Liu Y, Fu M, Ning J, Yu L, et al Prevalence of vitamin D insufficiency among children in southern china: A cross-sectional survey. Medicine. 2018;97(25):e11030 Epub 2018/06/21. 10.1097/MD.0000000000011030 . 29923990 PMC6023856

[16] Shu-Chi M, Jiann-Loung H, Yu-Hung L, Tseng-Chen S, Ming IL, Tsu-Fuh Y. Growth and development of children conceived by in-vitro maturation of human oocytes. Early human development. 2006;82(10):677–82. Epub 2006/05/13. 10.1016/j.earlhumdev.2006.01.012 . 16690233

[17] Adela R, Borkar RM, Bhandi MM, Vishwakarma G, Reddy PN, Srinivas R, et al Lower Vitamin D Metabolites Levels Were Associated With Increased Coronary Artery Diseases in Type 2 Diabetes Patients in India. Scientific reports. 2016;6:37593 Epub 2016/11/25. 10.1038/srep37593 27883024 PMC5121614

[18] Bikle DD. Vitamin D metabolism, mechanism of action, and clinical applications. Chemistry & biology. 2014;21(3):319–29. Epub 2014/02/18. 10.1016/j.chembiol.2013.12.016 24529992 PMC3968073

[19] Kongsbak M, Levring TB, Geisler C, von Essen MR. The vitamin d receptor and T cell function. Frontiers in immunology. 2013;4:148 Epub 2013/06/21. 10.3389/fimmu.2013.00148 23785369 PMC3684798

[20] Altamirano-Barrera A, Barranco-Fragoso B, Mendez-Sanchez N. Management strategies for liver fibrosis. Annals of hepatology. 2017;16(1):48–56. Epub 2017/01/05. 10.5604/16652681.1226814 . 28051792

[21] Ding N, Liddle C, Evans RM, Downes M. Hepatic actions of vitamin D receptor ligands: a sunshine option for chronic liver disease? Expert review of clinical pharmacology. 2013;6(6):597–9. Epub 2013/10/30. 10.1586/17512433.2013.841078 24164608 PMC4160153

[22] Dong R, Sun S, Liu XZ, Shen Z, Chen G, Zheng S. Fat-Soluble Vitamin Deficiency in Pediatric Patients with Biliary Atresia. Gastroenterology research and practice. 2017;2017:7496860 Epub 2017/07/12. 10.1155/2017/7496860 28690638 PMC5485346

[23] Shneider BL, Magee JC, Bezerra JA, Haber B, Karpen SJ, Raghunathan T, et al Efficacy of fat-soluble vitamin supplementation in infants with biliary atresia. Pediatrics. 2012;130(3):e607–14. Epub 2012/08/15. 10.1542/peds.2011-1423 22891232 PMC3428752

[24] Lee WS, Jalaludin MY, Wong SY, Ong SY, Foo HW, Ng RT. Vitamin D non-sufficiency is prevalent in children with chronic liver disease in a tropical country. Pediatrics and neonatology. 2018 Epub 2018/04/24. 10.1016/j.pedneo.2018.03.011 . 29680189

[25] Jensen M, Abu-El-Haija M, Bishop W, Rahhal RM. Difficulty Achieving Vitamin D Sufficiency With High-Dose Oral Repletion Therapy in Infants With Cholestasis. Journal of pediatric gastroenterology and nutrition. 2015;61(2):187–9. Epub 2015/02/05. 10.1097/MPG.0000000000000751 . 25651487

[26] Houghton LA, Gray AR, Harper MJ, Winichagoon P, Pongcharoen T, Gowachirapant S, et al Vitamin D status among Thai school children and the association with 1,25-Dihydroxyvitamin D and parathyroid hormone levels. PloS one. 2014;9(8):e104825 Epub 2014/08/12. 10.1371/journal.pone.0104825 25111832 PMC4128742

